# Evaluation of BioCreAtIvE assessment of task 2

**DOI:** 10.1186/1471-2105-6-S1-S16

**Published:** 2005-05-24

**Authors:** Christian Blaschke, Eduardo Andres Leon, Martin Krallinger, Alfonso Valencia

**Affiliations:** 1 Protein Design Group, National Center of Biotechnology, CNB-CSIC, Cantoblanco, E-28049 Madrid, Spain; 2 Bioalma SL, Ronda de Poniente 4- 2nd floor, Tres Cantos, E-28760, Madrid, Spain

## Abstract

**Background:**

Molecular Biology accumulated substantial amounts of data concerning functions of genes and proteins. Information relating to functional descriptions is generally extracted manually from textual data and stored in biological databases to build up annotations for large collections of gene products. Those annotation databases are crucial for the interpretation of large scale analysis approaches using bioinformatics or experimental techniques. Due to the growing accumulation of functional descriptions in biomedical literature the need for text mining tools to facilitate the extraction of such annotations is urgent. In order to make text mining tools useable in real world scenarios, for instance to assist database curators during annotation of protein function, comparisons and evaluations of different approaches on full text articles are needed.

**Results:**

The Critical Assessment for Information Extraction in Biology (BioCreAtIvE) contest consists of a community wide competition aiming to evaluate different strategies for text mining tools, as applied to biomedical literature. We report on task two which addressed the automatic extraction and assignment of Gene Ontology (GO) annotations of human proteins, using full text articles. The predictions of task 2 are based on triplets of *protein – GO term – article passage*. The annotation-relevant text passages were returned by the participants and evaluated by expert curators of the GO annotation (GOA) team at the European Institute of Bioinformatics (EBI). Each participant could submit up to three results for each sub-task comprising task 2. In total more than 15,000 individual results were provided by the participants. The curators evaluated in addition to the annotation itself, whether the protein and the GO term were correctly predicted and traceable through the submitted text fragment.

**Conclusion:**

Concepts provided by GO are currently the most extended set of terms used for annotating gene products, thus they were explored to assess how effectively text mining tools are able to extract those annotations automatically. Although the obtained results are promising, they are still far from reaching the required performance demanded by real world applications. Among the principal difficulties encountered to address the proposed task, were the complex nature of the GO terms and protein names (the large range of variants which are used to express proteins and especially GO terms in free text), and the lack of a standard training set. A range of very different strategies were used to tackle this task. The dataset generated in line with the BioCreative challenge is publicly available and will allow new possibilities for training information extraction methods in the domain of molecular biology.

## Background

The recent advances in Molecular Biology are responsible for the accumulation of various and complex data types. They include biological sequences derived from genome projects, and structural data of biomolecules from the structural genomic initiatives. One of the more important items is the characterization of protein function obtained through biochemical and genetic experiments. To handle the increasing amount of complex data, computational methods are being developed in the areas of bioinformatics and computational biology.

A number of comparative assessments of the different computational approaches, addressing not only independent evaluation of resources but also the accessibility of the tools for real world applications have been carried out.

The *Critical Assessment of Protein Structure Prediction *(CASP) contest constitutes one of the first community wide experiments to benchmark the state of the art of protein structure prediction (refer to Proteins. 2003;53 Suppl 6:524-33). CASP has been running for a decade and had served as a model for later initiatives. Among those initiatives are the *Critical Assessment of Microarray Data Analysis *(CAMDA) contest to analyze the performance of microarray bioinformatics tools [1] and the *Critical Assessment of PRediction of Interactions *(CAPRI) contest for the assessment of protein interaction prediction techniques [2].

Also for genome bioinformatics an evaluation contest was carried out, called *Genome Annotation Assessment Project *(GASP) [3]. Other assessments of computational tools applied to the biomedical domain include the *Genome Access Workshop *(GAW) for statistical genetics techniques [4] and the *Predictive Toxicology Challenge *(PTC) for computational toxicology approaches [5].

The biomedical literature constitutes one of the most valuable data sources for functional descriptions of biomolecules, and as such it is constantly subject to manual extraction of relevant information by biological database curators as well as by individual researchers. Given the volume of publications and functional descriptions, a number of computational analysis techniques have been developed in recent years to extract information from biological text sources.

The community-wide evaluation strategies are not exclusive to the bioinformatics domain, they are also used commonly to estimate the performance of information extraction and retrieval tools, e.g. the Message Understanding Conferences (MUCs) [6].

In the domain of biomedical literature, the knowledge discovery and data mining (KDD) challenge cup [7] evaluated how text mining tools could aid in the process of database curation, in this case of the FlyBase database [8]. The first Genomics track [9] of the Text REtrieval conference (TREC) focused on the evaluation of current strategies of ad hoc retrieval and information extraction of biomedical texts. The *Critical Assessment for Information Extraction in Biology *(BioCreAtIvE) contest was organized to evaluate current text mining techniques applied to the biological research literature in biologically realistic scenarios, including the evaluation of different text mining approaches aimed to solve two tasks focused on the use of information by biologists and database curators. The two major tasks addressed by this contest were the extraction of gene names and the normalization of genes [10,11], while the second task, which will be discussed in detail in this article, was the extraction of protein annotations from full text scientific articles. The assessment was discussed in the context of a workshop held in March 2004 (refer to ).

### Task 2 description

Gene Ontology (GO) provides a consistent set of controlled vocabularies (concepts) which are useful to annotate gene products, such as proteins [12]. The terms organized in GO are nowadays the most important biological annotation resource and display a range of advantages over previous annotation efforts based on functional keywords. There are three main categories used to describe relevant aspects of gene products, namely *cellular component*, *biological process *and *molecular function*. These relevant biological aspects of gene products are extensively used to annotate proteins within biological databases (e.g. GOA) [13]. Therefore GO terms were considered for task 2 of the BioCreAtIvE contest, addressing the assignment of functional annotations (GO terms) to human gene products using text mining and information extraction techniques. The training and test set for annotations using GO terms were provided by human experts (GOA curators) who are involved in the manual assignment of GO terms to gene products [14]. The analyzed annotations were extracted from full text articles, because often the annotation-relevant text passages, and especially the experimental evidence supporting those annotations, are not provided in the abstracts accessible in PubMed. Task 2 was divided into sub-tasks each focusing on certain aspects associated with the annotation process. A total of nine teams participated at task 2; each group could submit up to three results for each single run. More than 15,000 individual results were submitted for evaluation by a team of three curators, who dedicated several month to the evaluation of the results [14].

### Task 2.1 Identification of annotation relevant text passages

The aim of sub-task 2.1 was to evaluate different approaches for the extraction of text passages which contain statements that relate functional annotations for GO terms to the corresponding gene products. The participating systems were provided with a test set consisting of triplets of protein identifiers (Swiss-Prot accession number), GO identifiers and the articles' filenames. Then they returned text fragments which contain predictions consisting of information relevant to the annotations of the corresponding GO term and associated gene products. The assessment did not specify any explicit length of the evidence text.

### Task 2.2 Assignment of GO terms to gene products

The purpose of sub-task 2.2 resembled the typical human annotation procedure, in the sense that the participants had to return the annotations derivable from a given protein-article pair. The annotations which are contained within the articles should thus be automatically identified and the corresponding GO-term returned together with the supporting text passage. In order to make this task easier, the number of protein-GO term associations for each GO category contained in each article was provided for the test set (see data set section).

### Task 2.3 Selection of relevant papers

Within this sub-task, given a collection of articles, those papers should be returned which are relevant for the annotation of certain proteins to derive GO annotations for them. Also the evidence text fragments should be returned. In this sub-task, the groups were asked, given a collection of articles, to return papers relevant for the annotation of certain proteins together with the GO annotations and the text fragment evidence. The evaluation of subtask 2.3, an ad hoc retrieval task, was not carried out in the current BioCreAtIvE evaluation. A similar task was posed at the TREC Genomics track 2004 [15].

## Data set and evaluation strategy

The Gene Ontology Annotation (GOA) database  provides a large collection of manually extracted associations of proteins to GO terms. Curators responsible for those annotations have a high degree of expertise in carefully annotating proteins with their corresponding functional and biological information. Therefore the GOA curators at the European Bioinformatics Institute (EBI) were asked to evaluate the results of automatic annotation extraction tools that took part in the BiocCreAtIvE task 2 [14]. The GOA database contains manually extracted associations of proteins to GO terms, providing the article identifier which contains the information source for the annotation itself, as well as the type of evidence supporting those annotations [13].

For instance the following example corresponds to a single GOA entry:

P41220 RGS2_HUMAN GO:0005096 PMID:10747990 TAS F Regulator of G-protein signaling 2 IPI00013177.

Here the protein with the accession number P41220 has been annotated as a 'Regulator of G-protein signaling 2' (GOID 0005096) using information derived from the article with the PubMed ID '10747990'. For the assessment itself, three distinct expert annotators were responsible for the evaluation of the submitted predictions. This allowed an estimate of inter-annotator agreement and objective evaluation metrics [14].

### Data preparation: the training data

As already mentioned the training data encompassed basically GOA annotations and the GO terms as well as full text articles. Although GOA provides the associations and the corresponding article identifier, it doesn't contain a protein dictionary, and often the annotated protein appears in the textual data as a synonym or typographical variant which is not covered by the Swiss-Prot database. As we did not provide a fixed name dictionary for the contest, participants could use external publicly available sources which were suitable to cross-link the given protein to additional information such as synonyms or protein descriptions contained in databases like LocusLink [16] or HUGO [17]. Some participants integrated such additional information sources into their systems. The articles linked through GOA to the annotations are often only accessible as abstracts, as most of the journals do not provide free access to the full text articles. In practice the curators use full text articles for their annotation procedure, especially to support annotations based on experimental evidence. Taking only the abstract is often not enough to recover annotation relevant text passages.

GO annotations are associated with evidence codes, which are assigned to describe the type of evidence used to create the annotations . We did not make use of the following evidence codes, because these annotations cannot be retrieved from the literature: IC (based on curator judgment), ND (no data) and IEA (inferred from electronic annotation).

The terms which build up GO are categorized into three non-overlapping branches: Cellular Component, Molecular Function and Biological Process. A protein may be annotated with one or more terms from each category, related to information that appears in many different articles. As the curators follow a protein centered approach, those articles might contain additional functional annotation for other proteins which are not used in GOA.

The GOA release of May 2003 was used for this experiment; it contained approximately 84604 annotations. A total of 9725 PMIDs were used to derive annotations. The corresponding articles were further processed to select those which corresponded to the *Journal of Biological Chemistry *(JBC) (a total of 1683). As we had access only to a certain release of the JBC articles, those which corresponded to an available full text article were selected. A set of 640 JBC articles remained which had linked GO annotations provided by GOA. Also a number of full text articles belonging to the journals *Nature Medicine*, *Nature Genetics *and *Oncogene *were filtered in a similar way to obtain only those articles used for GO annotations as provided in GOA (163 articles). The final training set thus contained a total of 803 full text articles from four different journals that were provided in Standard General Markup Language (SGML) format.

The provided training set constituted a data source which provides only indirect linkage between text passages and protein-GO annotations and not the direct passages of text in which the GO-protein relation can be found. Although this adds difficulty to the training of various computational systems based on learning techniques (AI), we think that it reflects the real world scenario encountered by database curators.

### The test data

The BioCreAtIvE test set contained full text articles, just as the database annotators use for their work. A total of 212 full text articles freely distributed by the *Journal of Biological Chemistry *(JBC) in SGML format were provided to the participants, 113 for task 2.1 and 99 for task 2.2. Those articles were dated between the years 1998 and 2002. The GOA curators provided a total of 1076 gene product-journal-GO term associations to the participants for task 2.1, such as *O75612 JBC_1998-2/bc028208.gml 0005515*, where *O75612 *corresponds to the protein accession number from Swiss-Prot, *JBC_1998-2/bc028208.gml *is the name of the file containing the article and *0005515 *the GOID of the term which has been manually annotated to the protein. In case of task 2.2, the test data contained for each protein and journal pair, the number of annotations per GO-category encountered by the curators in the article. For instance the protein with the Swiss-Prot accession number *P16471 *had 7 biological process terms, 1 cellular component term, and 2 molecular function terms associated through the article JBC_1999-2/bc035461.gml. For the task 2.3, the teams were asked to provide for, ten proteins, the articles which are relevant for annotation, together with the GO terms and the annotation text passages.

The numerical summary of the training and test sets used in task 2 is contained in table 1. When considering the overlap between the proteins used in the training set and the proteins appearing in the test set, 11 of them occur in both the training and the test set of task 2.1 and 8 in case of task 2.2. A total of 185 GO terms of the task 2.1 test set are also contained in the training set, while 165 GO terms of task 2.2 are also present in the training data. This means that only a fraction of GO terms both were present in the training and the test set.

### Evaluation strategy

The evaluation was carried out by three GOA database curators, see accompanying article [14]. The Extensible Markup Language (XML)-like submissions contained text fragments marked to allow the evaluators to decide whether the predictions were correct or not. In case of sub-part 2.2 also the prediction of the GO code itself was also assessed, together with text passage supporting the annotation. The text passages submitted as evidence by the various teams were highlighted by a tool to facilitate the evaluation. A substantial number of predictions were revised that were associated with randomly number of proteins (x), providing sufficient grounds for the statistical analysis of the results.

After revising the predictions, the GOA evaluators decided about the quality of the predictions by following the protein accession number and the GOID. Three levels of accuracy were used for the annotations by the evaluators including the evaluation of the presence of the GO term and/or corresponding proteins and verifying their relation with the submitted text passage. Also additional comments related to the predictions were provided by the curators regarding the quality of the predictions. The independent predictions for both GO terms and proteins were scored as *high *in cases where the protein or the GO term were extracted correctly. The submissions tagged with *generally *corresponded to those predictions which were generally correct, but too general to be of practical use. For instance in case of the protein predictions, this means that the specific protein was not identified but a homologue from another organism or a general reference to the corresponding protein family was encountered. In case of the GO term predictions scored as *generally*, a high level parent term of the actual GO term might be referenced. Results tagged as *low *are basically wrong predictions. A double identification in a given text passage of *high *for protein and GO term implies the correct (*high*) identification of the association between them. Concerning task 2.3, the limited number of participants and the technical difficulty of the evaluation did not allow us to assess the results of sub-task 2.3 in time for the assessment workshop.

## Results

The dataset produced at the BioCreative contest task two is freely available from: [18] and is given in as an XML-like format. From the nine registered users who participated in task 2.1, a total of 15,992 evidence passages were provided to the curators. Out of those, 12,014 corresponded to the requested queries (the rest corresponded to new predictions which were not contained in the test set). On average 11.34 (standard deviation of 2.30) submissions of annotation predictions were sent for each single query triplet across all the user submissions (21 runs). Users submitted between a single run up to the maximum of three runs allowed; there were 21 runs submitted for task 2.1. This was especially work-intensive for the GOA annotators (evaluators), as in many cases the textual passages returned were entire paragraphs. It is possible to distinguish between two approaches followed by the participants. The majority of users tried to submit a result for each case contained in the test set. Those approaches focused on obtaining a high recall rather than a high precision. On the other hand, there were users who submitted results only for a small number of high confidence predictions to achieve a high precision. Although for practical use of text mining applications, high precision is desirable, a reasonable recall is essential, consequently an compromise between both should be favored.

Of the diverse approaches adopted, three main strategies can be characterized.

1) Methods were centered in the GO terms themselves, and in general used pattern matching and matching of the words making up the GO terms; these were associated to a certain weight or frequency and part of speech information. Those approaches tried to submit results for each query and were thus centered in reaching high number of correct predictions. For instance *Couto et al*. [19] based their information extraction method on the calculation of the information content of each GO term. *Ehrler et al*. [20] applied manually crafted regular expressions or heuristic rules in their methods. A more computational linguistic approach was followed by *Verspoor et al*. [21] which incorporated statistical term frequency and 'part of speech' information. Finally *Krallinger et al*. [22] constructed a heuristic weight scheme to words or terms associated with the original query GO term, matched to sentence windows.

2) Other strategies are characterized by the use of machine learning techniques. Due to the lack of a high quality training set, those strategies were less effective than others. Some of those methods use words co-occurring with GO terms to derive their training set. *Rice et al*. [23] applied term based support vector machines to return the paragraph which might contain the annotation relevant passages while *Ray et al*. [24] applied Naïve Bayes models and n-gram models to rank the paragraphs according to their annotation associations.

3) Finally the third tendency is characterized by the aim of reaching a high precision through pattern matching and template extraction. *Chiang et al*. [25] implemented a hybrid approach which focused on high precision. It is based on phrasal pattern matching and a sentence classification system using Naïve Bayes methods, as well as term indexing techniques. Although the obtained recall is low it achieved a high precision.

Table 2 lists the different features and resources used by the participants. Not only does the basic processing unit differ between the various approaches (e.g. sentence level vs. paragraph level) but also the methods themselves are diverse. Despite this variety within the procedures used, some commonalities among them can be identified. For instance, the majority of users worked at sentence level, and processed the full article. Almost half of the participants integrated a machine learning method into their approach. A significant number of the participants took advantage of pattern matching and regular expressions. Regarding external resources, the HUGO database and the UMLS/MeSH dictionary were used by two participants. An overall summary of the distinct participating groups is provided by table 3.

### Task 2.1

**Figure 1:**
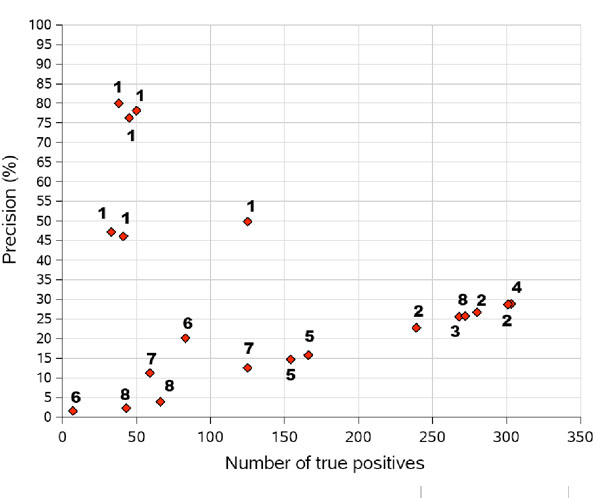
**Task 2.1 Precision versus total true positives (TP) plot. **Task 2.1 results: precision vs total number of true positives (protein and GO term predicted correctly, i.e. evaluated as 'high'). Each point represents a single run submitted by the participants of task 2.1. 1: Chiang et al., 2: Couto et al., 3: Ehrler et al., 4: Krallinger et al., 5: Krymolowski et al., 6:Ray et al., 7: Rice et al., 8: Verspoor et al.

The aim of sub-task 2.1 was to assess tools able to extract text fragments to support the annotation of a given protein-GO term association. Table 4 shows the overall results obtained for each run by the different groups and figure 1 shows the results in terms of TP (i.e. correct predictions) vs. precision. The group which obtained the highest precision results was *Chiang et al*. [25], with a precision of 0.80, although the number of correct predictions was of only 36 annotations. All the runs submitted by this group are characterized by high precision and low recall (ranging from a total of 45 to 251 submissions and precisions from 0.46 to 0.80). On average this group has also the highest percentage of overlap with respect to the correct predictions submitted by other groups. When considering the total number of correct predictions (TP), *Krallinger et al*. [22] (303 annotations) and *Couto et al*. [19] (301 annotations) obtained the highest number of correctly extracted GO-protein associations. Both groups obtained a very similar number of correct annotations and there is also a higher overlap between the correct predictions of those two groups when compared to others. The precision of these methods was rather low (0.29), as they submitted results for all queries. Both methods associate the GO terms and the word tokens forming the GO terms with a weight, a heuristic sub-tag weight in case of [22] and the information content in case of [19]. Other groups who extracted a large number of correct annotations were *Verspoor et al*. [21] and *Ehrler et al*. [20] with 272 and 268 correct predictions respectively and a precision of 0.26.

#### Gene ontology terms

Not only the evidence passages but also the identification of GO terms was assessed. The scoring scheme was, as already mentioned, divided into three sets of extraction accuracy (*high*, *generally *and *low*). True positive (TP) predictions were considered as those which were evaluated as high for both the GO term and the corresponding protein.

**Figure 2:**
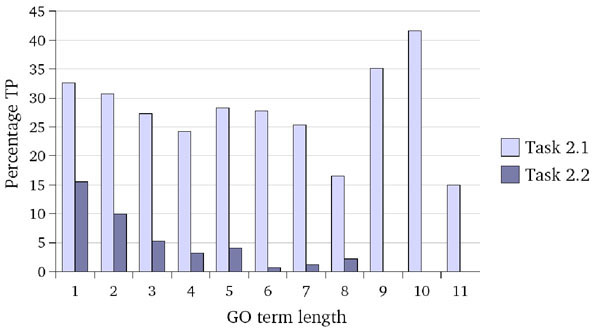
**Task 2.1 True positive predictions compared to GO term length. **This plot shows the association between the number of true positives (TP), meaning predictions where the GO term and the corresponding protein were correctly predicted (evaluated as 'high') and the length of GO terms in task 2.1. The length of GO terms was measured by the number of words which form the terms, after splitting at spaces, and certain special characters (e.g. '-', '/' and ',').

In general shorter GO terms, with lengths between 1 and 4 words, show the tendency that shorter ones are easier to predict than longer ones, and that the difficulty increases with the length of the term (see figure 2). This is similar to the case of gene names in task 1, where shorter gene names (e.g. yeast genes) are better extracted when compared to longer gene names (e.g. mouse genes). Nonetheless terms with a length of 5 words have an increased percentage of correct predictions. This could in part be explained by the presence of some information rich words in those GO terms. There is again a tendency that shorter terms are easier to predict than longer ones in the range of GO term lengths of between 5 to 8 words. The high percentage of correct predictions of GO terms with lengths of between 9 and 10 words are basically outliers. It is important to take into account that some words forming the GO term are stop words or unspecific words, and others are polysemic (they may have several meanings, and might thus be used in a different context, not associated to the sense provided in the GO term). GO terms which contain polysemic words, or words which are often used in a different context (e.g. as part of an experimental method) are more difficult to extract.

**Figure 3:**
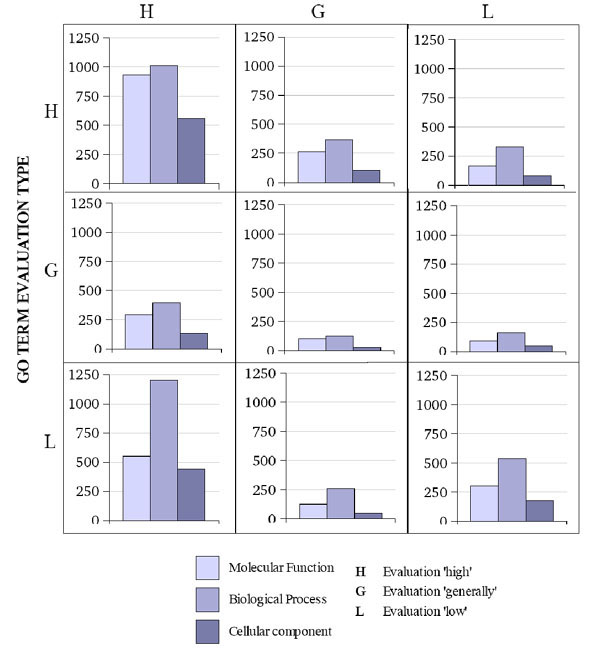
**Task 2.1 Evaluation of the annotation predictions depending on the GO categories. **Evaluation of task 2.1 prediction depending on the evaluation type and the GO categories. H: high (correct prediction), G: generally (overall correct but too general to be of practical use) and L: low (basically wrong predictions). The GO categories are CC: Cellular Component, MF: Molecular Function and BP: Biological Process. Notice that only the entirely evaluated predictions are displayed.

Also the predictions according to the distinct GO categories were analyzed in detail. Figure 3 shows the different evaluation types for the annotation predictions related to the three GO categories. When considering the set of evaluated submissions, GO annotations of the category Cellular Component had the highest fraction of correct (i.e. protein high and GO term high) predictions with 34.61 % (561 of 1621 evaluated annotations), followed by the Molecular Function category with 33.00% (933 of 2827) of correct annotations and Biological Process with only 23.02% (1011 of 4391) of correct annotation predictions. This correlates with the average length of the submitted GO terms, where Cellular Component terms had an average length of 2.03, Molecular Function terms an average length of 3.35 and Biological Process terms of 3.56. Thus in general the Cellular Component terms of the test set corresponded to short descriptive names compared to the longer and more complex terms of the Biological Process category.

#### Protein names

To extract correct annotations it is also important to identify the protein names and symbols in the articles. This was the main concern of task 1 of the BioCreAtIvE contest. In task 2, the participants were provided with Swiss-Prot accession numbers of human proteins rather than the protein names themselves, and as proteins usually appear in free text as symbols or names, they had to use links to databases such as UniProt or HUGO to obtain lists of protein names, symbols and descriptions. The tools used in task 1 performed in general significantly better when compared with the protein identification strategies used in task 2, as most of the participants focused on the identification of the GO term. The overall performance of protein identification was better than the GO term extraction, not only for sub-task 2.1 but especially for sub-task 2.2, meaning that it is easier to find text passages which refer to a given query protein than GO terms. The identification of the protein names is actually a variant of the named entity task, which is known to perform well, around 80 percent for the protein and genes in case of task 1A. A detailed analysis of the evaluation of the protein extraction is given in the BioCreAtIvE workshop handouts [18].

### Task 2.2

The sub-task 2.2 was concerned with automatically assigning GO terms to protein and article pairs, returning the text passages which support those assignments. Thus, it consists basically of a text categorization and passage retrieval task.

A total of 5258 predictions were submitted by the participants, which corresponded to 3882 unique protein-GO term-article triplets. A total of 4976 were completely evaluated (i.e. evaluation of both the protein and the GO term). Of the predictions submitted, 2392 terms belonged to the GO category Biological Process, 1811 to the Molecular Function category and 1055 to the Cellular Component category. The average number of submissions for a query triplet was 1.35. When considering the length of the GO terms measured as number of word tokens (after splitting at spaces and certain special characters such as hyphens, slashes and backslashes), the average length of the submitted terms was 2.81. Regarding the length of the submitted GO terms for the different GO categories, there were also differences depending on the GO categories. The average length of Molecular Function terms was 3.04 (standard deviation of 1.46), of Biological Process terms 3.09 (standard deviation of 2.28) and of Cellular Component terms 1.78 (standard deviation of 1.21). Thus the predicted terms of the Cellular Component category were generally shorter than the other categories. The total number of correct (true positive) predictions, evaluated as protein-high (correct) and GO term-high (correct) was 422 out of the 3882 evaluated submissions. The corresponding GO terms of the correct predictions had an average length of 1.92 (standard deviation of 1.06) while the overall average GO term length of all the evaluated predictions was of 2.81.

**Figure 4:**
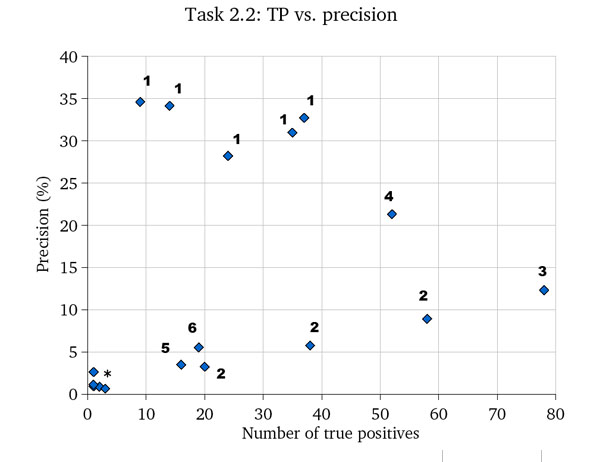
**Task 2.2 Precision versus total true positives plot. **Task 2.2 results: Precision vs. total number of True Positives. Each point represents a single run submitted by the participants of task 2.2. User 1: Chiang et al., 2: Couto et al., 3: Ehrler et al., 4: Ray et al., 5: Rice et al., 6: Verspoor et al., *: the remaining runs, refer to table 5.

When analyzing the evaluated submissions per GO category, perfect predictions of cellular component category constituted 11.04% of the evaluated predictions, with an average GO term length of 1.26. In the case of the Molecular Function category, 8.91% of the evaluated predictions were correct, with an average GO term length of 2.80. For the Biological Process category 7.04% of the evaluated predictions were correct (average GO term length of 2.54%). Considering the absolute number of correct (TP) predictions, Ehrler *et al. *[20] obtained the best performance (78 TP submissions) in run 1 (12.30%) followed by Couto *et al. *[19] with 58 (8.91%) TP predictions. Considering both precision and recall, Ray *et al*. [24] reached 52 correct predictions with a precision of 21.31%, which is considerably higher than in the case of Ehrler *et al. *and Couto *et al*. The participant which reached the highest precision was Chiang *et al. *[25] with 34.62% (9 out of 26 predictions) and 34.15% (14 out of 41) correct predictions (see figure 4 and table 5).

There are also predictions which are in principle correct, but the assigned GO term is too general to beuseful for practical purposes (evaluated as 'Generally).

## BioCreAtIvE corpus

The evaluation of the task 2 predictions was carried out by GOA database curators and was based on the returned evidence text. In case of sub-part 2.2, the prediction of the GO code itself was also assessed together, with the annotation text passage. The XML-like submissions contained thus text fragments critical for the evaluators to decide whether the predictions were correct. Those text passages were highlighted by a tool used by the evaluators to visualize the submitted text passages within the whole article. This visualization and text highlighting program was implemented for the evaluation team and facilitated the assessment of the submitted text passage within its context in the whole article. Therefore it helped to speed up the evaluation and provided a standard interface to assist the scoring of the submitted predictions. This was done, having in mind future practical applications using those predictions utilities. The data set produced during the BioCreAtIvE contest, i.e. the evaluated predictions, has been released and is freely accessible through the web [18]. It is provided in an XML-like format and contains tags which label the evaluation type for each prediction. To obtain the dataset, an agreement must be signed which contains the contact information and assures that the dataset will be used for research purposes only. The length of the evidence passages is highly variable, as some of the predictions consist of entire paragraphs, while other predictions consist only in a single sentence.

## Discussion

The use of GO terms for a text mining task was challenging because the terms which build up GO are controlled concepts which might be expressed in natural language text in a number of different ways. Moreover there are over 15,000 concepts in GO. GO is actively maintained and continually expanded. It constitutes a widely used set of terms for protein annotation, fulfilling the demands to support annotation in multiple biology databases, such as Swiss-Prot and UniProt.

Only the use of biologically inspired tasks for text mining tools will provide methods which are of practical relevance for biologists and bioinformaticians. The integration of bioinformatics applications with text mining tools might create new knowledge sources in the future. Community wide evaluations of biomedical text mining strategies can assist the process of improving currently available text mining and information extraction tools and speed up the integration of the heterogeneous data types produced in life sciences. A broad range of techniques were applied to extract the relation of GO term to proteins in text (task two). Among the main difficulties encountered in task two were the lack of a high quality training set consisting in the annotation relevant text passages rather than full text articles associated with certain protein-GO annotations. The overlap between GO terms in the training and the test set was also rather low, which especially effected approaches relying on machine learning techniques. The over-annotation of the test set (on average more GO terms were extracted by the GOA evaluation team from test set articles than was the case for the training set articles) reflected the article-centric approach in the test set versus the protein centered approach of the training set. Therefore GO terms which in case of GOA (training set) annotations might have been discarded were included in case of this challenge. The vast amount of existing GO terms (large number of classes), the lack of a substantial number of available synonyms for those GO terms and the use of full text articles rather than abstracts posed additional difficulties for the participants.

Although the number of GO terms which comprise the test sets of task two is small when compared to all GO terms, it is still useful in providing an insight into particular aspects of the three categories which build up GO. For instance when looking at the length of correctly predicted GO terms of sub-task 2.1, there was an inverse relation between the average length of the GO terms of each category and the percentage of correct predictions. This means that the terms belonging to the Cellular Component category are on average shorter (average length of 2.03 words) and contain more informative words and therefore were easier to detect (percentage of correct predictions was 34.61%) when compared, for instance, to the Biological Process terms (with average length of 3.56 words and a percentage of correct prediction of 23.02%).

The order of difficulty in predicting the terms belonging to each GO category is identical for sub-task 2.2. Although in general shorter terms seem to be easier to predict, this is not always the case when retrieving terms which are formed by a single word. We propose that when predicting those terms some of them are too general to be of practical use (task 2.2). There are also cases when retrieving those words, where they are used with a different meaning (polysemic) which does not correspond to the meaning which is provided for the GO term. Moreover they appear often as part of expressions in a different semantic context (task 2.1 and task 2.2).

There were groups which took part in task 2 who gave priority the recall (predictions for every query case) while others focused on precision (only predicting a relatively small number of high confidence cases). Although a trade-off between both would be desirable, the potential end users have to decide depending on the needs in each case, whether they are interested in recall, precision or f-score (i.e. balanced precision and recall). For instance proteins which are highly quoted in the literature might be a case for high precision demands, while sparsely quoted proteins might be a target for high recall methods.

In general the overlap between the predictions made by the different groups is relatively small (except in the case of *Chiang et al.*), especially in case of sub-task 2.2. This agrees with the diverse methodological approaches implemented by the participants. In task 2.1 (retrieving the terms) most of the correct predictions were made only between 1–3 times, and in task 2.2 (predicting the terms) the vast majority of the correct predictions were made only once. This implies that the features and methods exploited by a certain participant are useful only for certain scenarios, while in other situations, other properties adopted by different strategies might be advantageous. An approach which is able to efficiently integrate the characteristics used by the different methods into a single tool could increase the performance significantly. The dataset produced within task 2 serves as a 'weak labelled' training set for future applications, meaning that although the text passages and their corresponding evaluations are provided, the exact words relating to the protein entity, GO terms and the relationship are not especially highlighted.

## Conclusion

The BioCreAtIvE challenge for evaluation of text mining tools applied to biomedical literature was organized in two main tasks, the first related to the detection of protein and gene names and the second task was concerned with the extraction of protein annotations based on GO terms. The assessment of the submitted predictions for task 2 pointed out that there is still need for significant improvement to make the existing tools valuable for practical purposes, especially in sub-task 2.2. Thus, to monitor future improvements in this field, a similar set up in the context of future evaluations will be necessary. The data set derived from this challenge, which is freely available, might serve as a valuable training data for new text mining tools. The progress based upon the availability of such training data should be monitored through future contests, which in turn could provide new data resources.

The evaluations of large collections of predictions in this field is very expensive and time consuming and relies on the expertise of professional database curators such as the GOA team. There are also lessons learned from this edition of BioCreAtIvE which might improve future assessments, for instance a limitation to one or two runs per participant instead of three would facilitate the task of the curators who evaluated the predictions, as this process is specially work intensive. Limitation on the length of the evidence passage could also reduce the workload of the curators assessing the evidence passages. Also two variants of submission types could be adopted in future tasks, in analogy to task 1. For instance a closed submission type would allow only the use of previously specified external resources, while an open submission type might also integrate other additional information resources or databases. In this way a comparison between the distinct methods would be easier. The future extension of GO itself in terms of an enriched lexicon of synonyms for GO terms is perhaps more suitable for NLP strategies. This use of such resources mightincrease the importance of text mining applications in the near future.

## Authors' contributions

CB and AV organized and coordinated task 2, organized the workshop and supervised the analysis of the evaluation of the results. EAL managed the training and test data, was responsible for the webpage and availability of the data submissions and implemented the text highlighting tools. MK assisted in the post-workshop data analysis. AV, MK and CB authored the article.

## Figures and Tables

**Table 1:** Task 2 dataset description in numbers. The table shows the basic numbers referring to the task 2 training and test datasets. The full text articles of the training set were from the *Journal of Biological Chemistry *(JBC), *Nature Medicine*, *Nature Genetics *and *Oncogene*, while the test set articles were all from JBC.

Data set description	Training set	Test set 2.1	Test set 2.2	Data Type
Full text articles	803	113	99	free text
Total of GO annotation	2317	1076	1227	annotations
Number of proteins in the GO annotations	939	138	138	proteins
Number of GO terms used in the GO annotations	776	580	544	GO terms
Average number of annotations per protein	2.467	7.797	8.891	annotations
Annotations of Molecular Function GO terms	709	330	356	annotations
Annotations of Biological Process GO terms	1061	544	701	annotations
Annotations of Cellular Component GO terms	547	182	170	annotations
Molecular Function terms in the annotations	343	173	179	GO terms
Biological Process terms in the annotations	339	334	314	GO terms
Cellular Component terms in the annotations	94	57	51	GO terms

**Table 2:** Main features used by the participating teams. The table shows the features and strategies adopted by the different participants and the number of users.

Characteristics (C), resources (R) and methods (M)	Users
(C) Sentence level (retrieval unit)	[19,20,22,25,26]
(C) Paragraph level (retrieval unit)	[21,23,24]
(C) Full article processed	[19,21,22,24,25]
(C) Full article processed except methods section	[26]
(C) Only abstract processed	[20]
(C) GO term – Protein distance	[22,24,25]
(M) Stemming	[20,22,24,26]
(M) POS tagging	[25,26]
(M) Shallow parsing	[25]
(M) Finite state automata	[20,25]
(M) Edit distance ranking	[20]
(M) Vector space model	[20,21]
(M) Machine learning technique	[23-25]
(M) Support Vector Machines	[23]
(M) Naïve Bayes models	[24,25]
(M) N-gram models	[24]
(M) External resource – tool: GATE NLP tool	[21]
(M) External resource – tool: Morphological normalizer BioMorpher	[21]
(M) External resource – tool: qtile query based ranking tool	[26]
(M) External resource – tool: Grok POS tagger	[25]
(M) Heuristic rules	[22,24-26]
(M) Regular expressions/pattern matching	[19,20,22,24,25]
(M) Literal string matching	[22,24]
(R) Protein name aliases (link to external databases)	[22,24,26]
(R) GO terms used	[19-26]
(R) GOA data used	[22-24]
(R) GO term forming words/tokens	[19,22,24,26]
(R) GO term variants	[22,25]
(R) External resource – data: Dictionary of suffixes	[24]
(R) External resource – data: UMLS/MeSH dictionary	[20,24]
(R) External resource – data: HUGO database	[22,24,26]
(R) External resource – data: SGD database	[24]
(R) External resource – data: MGI database	[24]
(R) External resource – data: RGD database	[24]
(R) External resource – data: TAIR database	[24]
(R) External resource – data: Procter and Gamble protein synoyms	[21]

**Table 3:** Task 2 participants. The table shows the overview of the participants undertaking task 2.

Participant	Methods	Task 2.1	Task 2.2
Ehrler *et al. *[20]	Sequentially applied finite state automata	Yes	Yes
Couto *et al. *[19]	Information content of terms	Yes	Yes
Krymolowski *et al. *[26]	Heuristic rules, query expansion and question answering system	Yes	No
Verspoor *et al. *[21]	Word proximity networks approach	Yes	Yes
Krallinger *et al. *[22]	Heuristic weight and sentence sliding window	Yes	No
Rice *et al. *[23]	Term-based SVM approach	Yes	Yes
Ray *et al. *[24]	Statistical learning/Naïve Bayes method	Yes	Yes
Chiang *et al. *[25]	Hybrid method: pattern matching and sentence classification.	Yes	Yes

**Table 4:** Task 2.1 results. The table shows the results of task 2.1 for each participant.

Participant	Run	Evaluated results	Perfect prediction	Correct protein/general GO
Ehrler *et al. *[20]	1	1048	268 (25.57%)	74 (7.06%)
Krymolowski *et al. *[26]	1	1053	166 (15.76%)	77 (7.31%)
Krymolowski *et al. *[26]	2	1050	166 (15.81%)	90 (8.57%)
Krymolowski *et al. *[26]	3	1050	154 (14.67%)	86 (8.19%)
Verspoor *et al. *[21]	1	1057	272 (25.73%)	154 (14.57%)
Verspoor *et al. *[21]	2	1864	43 (2.31%)	40 (2.15%)
Verspoor *et al. *[21]	3	1703	66 (3.88%)	40 (2.35%)
Chiang I *et al. *[25]	1	251	125 (49.80%)	13 (5.18%)
Chiang I *et al. *[25]	2	70	33 (47.14%)	5 (7.14%)
Chiang I *et al. *[25]	3	89	41 (46.07%)	7 (7.87%)
Chiang II *et al. *[25]	1	45	36 (80.00%)	3 (6.67%)
Chiang II *et al. *[25]	2	59	45 (76.27%)	2 (3.39%)
Chiang II *et al. *[25]	3	64	50 (78.12%)	4 (6.25%)
Krallinger *et al. *[22]	1	1050	303 (28.86%)	69 (6.57%)
Rice *et al. *[23]	1	524	59 (11.26%)	28 (5.34%)
Rice *et al. *[23]	2	998	125 (12.53%)	69 (6.91%)
Ray *et al. *[24]	1	413	83 (20.10%)	19 (4.60%)
Ray *et al. *[24]	2	458	7 (1.53%)	0 (0.00%)
Couto *et al. *[19]	1	1048	301 (28.72%)	57 (5.44%)
Couto *et al. *[19]	2	1048	280 (26.72)	60 (5.73%)
Couto *et al. *[19]	3	1050	239 (22.76)	59 (5.62%)

**Table 5:** Task 2.2 results. The table shows the results of task 2.1 for each user.

Participant	Run	Evaluated results	Perfect prediction	Correct protein/general GO
Ehrler *et al. *[20]	1	634	78 (12.30%)	49 (7.73%)
Verspoor *et al. *[21]	1	110	1 (0.91%)	1 (0.91%)
Verspoor *et al. *[21]	2	344	19 (5.52%)	9 (2.62%)
Verspoor *et al. *[21]	3	229	2 (0.87%)	10 (4.37%)
Chiang I *et al. *[25]	1	26	9 (34.62%)	3 (11.54%)
Chiang I *et al. *[25]	2	41	14 (34.15%)	1 (2.44%)
Chiang I *et al. *[25]	3	41	14 (34.15%)	1 (2.44%)
Chiang II *et al. *[25]	1	113	35 (30.97%)	8 (7.08%)
Chiang II *et al. *[25]	2	85	24 (28.24%)	6 (7.06%)
Chiang II *et al. *[25]	3	113	37 (32.74%)	11 (9.73%)
Rice *et al. *[23]	1	479	3 (0.63%)	8 (1.67%)
Rice *et al. *[23]	2	460	16 (3.48%)	26 (5.65%)
Ray *et al. *[24]	1	244	52 (21.31%)	23 (9.43%)
Ray *et al. *[24]	2	38	1 (2.63%)	0 (0.00%)
Ray *et al. *[24]	3	90	1 (1.11%)	1 (1.11%)
Couto *et al. *[19]	1	617	20 (3.24%)	30 (4.86%)
Couto *et al. *[19]	2	661	38 (5.75)	26 (3.93%)
Couto *et al. *[19]	3	651	58 (8.91)	27 (4.15%)
